# Spoilt for choice: different immunosuppressive potential of anaplastic lymphoma kinase inhibitors for non small cell lung cancer

**DOI:** 10.3389/fimmu.2023.1257017

**Published:** 2023-09-26

**Authors:** Annkristin Heine, Stefanie Andrea Erika Held, Solveig Nora Daecke, Chrystel Flores, Peter Brossart

**Affiliations:** Medical Clinic III for Oncology, Hematology, Immuno-Oncology and Rheumatology/Clinical Immunology, University Hospital Bonn, Bonn, Germany

**Keywords:** alectinib, crizotinib, dendritic cells, immunosuppression, anaplastic lymphoma kinase (ALK) inhibitor, non-small cell lung cancer

## Abstract

**Introduction:**

Several anaplastic lymphoma kinase (ALK)-inhibitors (ALKi) have been approved for the treatment of ALK-translocated advanced or metastatic Non Small Cell Lung Cancer (NSCLC), amongst crizotinib and alectinib. This forces physicians to choose the most suitable compound for each individual patient on the basis of the tumor´s genetic profile, but also in regard to toxicities and potential co-treatments. Moreover, targeted therapies might be combined with or followed by immunotherapy, which underlines the importance to gain detailed knowledge about potential immunomodulatory effects of these inhibitors. We here aimed to 1.) determine whether ALKi display an immunosuppressive effect on human dendritic cells (DCs) as important mediators of antigen-specific immunity and to 2.) dissect whether this immunosuppression differs among ALKi.

**Methods:**

We investigated the effect of alectinib and crizotinib on human monocyte-derived DCs (moDC) as most powerful antigen-presenting cells. We performed immunophenotyping by flow cytometry, migration, antigen uptake and cytokine assays.

**Results:**

Crizotinib-treated DCs showed reduced activation markers, such as CD83, decreased chemokine-guided migration, lower antigen uptake and produced inferior levels of pro-inflammatory cytokines, especially Interleukin-12. In contrast, the immunosuppressive potential of alectinib was significantly less pronounced. This indicates that crizotinib might profoundly dampen anti-tumor immunity, while alectinib had no unfavourable immunosuppressive effects.

**Conclusions:**

Our results implicate that current ALKi differ in their capacity to suppress the activation, migration and cytokine production of DCs as essential mediators of T cell immunity. We show that crizotinib, but not alectinib, had immunosuppressive effects on DCs phenotype and reduced DC function, thereby potentially impairing anti-tumor immunity.

## Highlights

The 1^st^ generation anaplastic lymphoma kinase *inhibitor crizotinib deeply suppresses* dendritic cell differentiation, phenotype and function while the second generation anaplastic lymphoma kinase *inhibitor* alectinib has no suppressive effects on dendritic cells and T cell activation.

## Introduction

In the course of the detailed molecular and phenotypic profiling of NSCLC, the landscape of therapeutic options has been extended from surgery, chemotherapy and radiation to immunotherapy, targeted therapies and combined treatment approaches. Advanced, anaplastic lymphoma kinase (ALK) – positive Non Small Cell Lung Cancer (NSCLC) defines a specific molecular subtype among NSCLCs which is nowadays regularly treated with ALK inhibitors (ALKi). Different ALKi have recently been approved, comprising the first-generation ALKi crizotinib, the even more potent and selective second-generation ALKi alectinib, ceritinib, brigatinib and third generation ALKi lorlatinib (1–6). All inhibitors induce significant improvements in objective response rates (ORR) and progression-free survival (PFS) compared to chemotherapy in randomized phase I-III trials (7, 8). Second-generation ALKi might overcome crizotinib-resistant ALK mutations and have shown high overall response rates (ORR) (48% - 71%) in crizotinib-resistant patients and even higher ORRs in untreated patients (9). They are also effective in the absence of crizotinib-resistant *ALK* mutations, likely reflecting incomplete inhibition of ALK by crizotinib in some cases (9, 10). Thus, second or third line ALKi represent the current gold-standard for first-line treatment for ALK-rearranged metastatic NSCLC.

However, most patients on ALKi will eventually progress and require further systemic therapy. Combining ALKi with chemotherapeutic approaches has been proven successful in recent studies of multiagent therapy (11). In this context, a detailed knowledge on possible immunomodulatory effects is essential, as immunosuppression by ALKi could also hamper programmed death (PD-1)/programmed death ligand 1 (PD-L1) targeted CPI or other immunotherapeutic approaches in later therapy lines. Kleczko et al. recently reported that durable responses to alectinib in murine models of EML4-ALK lung cancer requires adaptive immunity (12). Although the clinical efficacy of cancer therapies heavily relies on the presence and induction of anti-cancer immune responses, only few reports exist on the immunomodulatory potential of ALKi:

The tumor microenvironment of ALK-positive NSCLC has been demonstrated to be immunosuppressive (13). It has been reported that Crizotinib can stimulate immunogenic cell death (14, 15), which might mediate the induction of potent anti-tumor immune responses. Further, ALKi and RET inhibitors can induce upregulation of human leukocyte antigen (HLA), can uncover tumor-associated antigens and decrease checkpoint molecules (16). It has been shown that driver mutations in general can increase PD-L1 expression and that over-expression of ALK fusion protein induces upregulation of PD-L1 (17, 18). ALKi-resistant cell lines displayed even higher levels of PD-L1 (18). In consequence, applying ALKi resulted in down-regulation of PD-L1. Another study found that TKIs, amongst Crizotinib, can activate the Nucleotide-binding oligomerization domain (NOD) -like receptor (NLRP3) inflammasome in myeloid cells (19). All these mechanisms help to explain how ALKi modulate anti-tumor immunity. However, differences amongst their immunoregulatory potential have not been elucidated in detail.

Efficient induction and expansion of T-cell mediated immunity is essential for the successful elimination of tumor cells. CPI enhance T-cell immunity and overcome T cell exhaustion. CD8 T+ cell induction requires professional antigen-presenting cells (APCs), mainly DCs, which present a specific antigen in presence of costimulatory molecules to naïve CD8+ T cells resulting in the induction of cytotoxic T cells responses (20). Given that DCs represent the most important players in the induction of antigen-specific immunity, an impaired DC function not only increases the risk for infections, but also diminishes anti-tumor immunity, likely resulting in worse therapy outcomes.

Due to the variety of approved ALKi for NSCLC, physicians are faced to choose the most suitable ALKi for each individual patient. Potential immunomodulatory effects of distinct ALKi could codetermine this choice. In this study, we therefore investigated whether first and second generation ALKi exert immunosuppressive effects on human DCs and questioned whether their immunosuppressive potential differs among miscellaneous ALKi.

## Methods

### Media and reagents

Human cells were cultured in RPMI 1640 containing glutamax-I, supplemented with 10% inactivated fetal calf serum (FCS) (RP10 medium) and 1% penicillin/streptomycin (Invitrogen), and treated with crizotinib and alectinib (Selleckchem) every other day in the indicated concentrations. These concentrations range from 0.1 to 1 μM, and correspond to serum levels achieved in treated patients.

### Generation of DCs

PBMCs were isolated by Ficoll/Paque (Biochrom, Berlin, Germany) density gradient centrifugation of blood obtained from buffy coats of healthy volunteers from the blood bank of the University of Bonn. The study was conducted in accordance with the declaration of Helsinki. Approval was obtained from the institutional ethics committee of the University Hospital of Bonn (#173/09).

1 × 10^8^ cells were seeded into 75 cm^2^ cell culture flasks (BD-Falcon, Heidelberg, Germany) in RPMI 1640 medium (see above). After 2 hours of incubation at 37°C/5% CO_2_, non-adherent cells were removed and cryopreserved at −80°C to be used for later analyses. Adherent cells were treated with human recombinant granulocyte macrophage-colony stimulating factor (GM-CSF; 100 ng/ml; Leukine Liquid Sargramostim; Bayer HealthCare Pharmaceuticals, USA) and interleukin-4 (20 ng/ml; R&D Systems, Wiesbaden-Nordenstadt, Germany) every second day starting at day 0 of culture as previously described (21). Maturation was induced on day 6 by adding LPS. Apoptosis of DCs was detected by live-dead staining using the propidium iodide kit from eBioscience. DCs were stained for flow cytometry analyses using commercially available monoclonal antibodies from BD Biosciences, Immunotech, R&D Systems, and eBioscience.

### *In vitro* migration assay

A total of 1 × 10^5^ cells were seeded into a transwell chamber (8 μm, BD Falcon; BD Biosciences) in a 24-well-plate. Migration towards chemokine (C-C motif) ligand (CCL)19 was analyzed after 4 hours by counting gated DCs for 1 minute by flow cytometry.

### Determination of cytokine production

Cytokine secretion was analyzed in the supernatant as indicated in the figures using the eBioscience™ ProcartaPlex Human Th1/Th2 Cytokine-Panel (Thermofisher) according to the manufacturer´s instructions.

### Statistical analysis

All experiments were performed at least 3 times, with representative experiments shown. To analyze statistical significance one-way analysis of variance (ANOVA) and Dunnett’s using Prism 8.4.3 software (Graphpad Software) were applied.

## Results

### Crizotinib impairs the differentiation of human monocytes into moDCs and inhibits proper moDC maturation upon TLR-ligation

**Figure 1:**
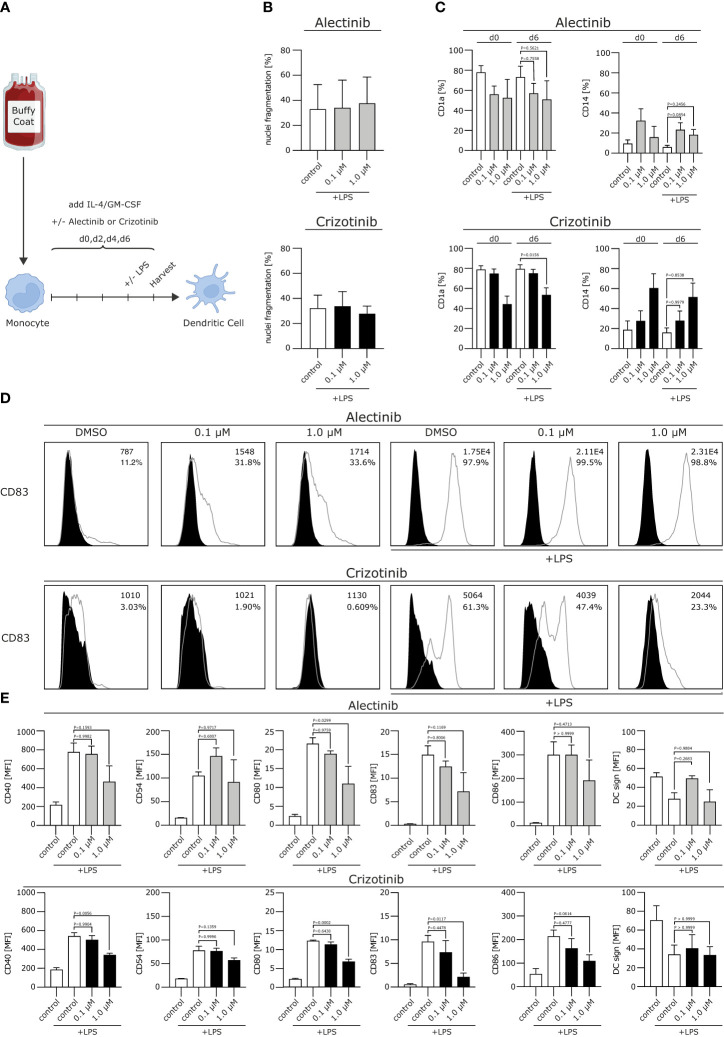
Crizotinib impairs the differentiation of human monocytes into moDCs and inhibits proper moDC maturation upon TLR ligation. **(A)** Schematic experimental design for the differentiation of moDCs from human monocytes. Monocytes were cultured under DC-driving conditions, using IL-4/GM-CSF. Alectinib, crizotinib or the control DMSO were added every other day in serum-equivalent concentrations (0.1 or 1 µM on day 0, 2, 4, 6). Cells were stimulated with TLR-ligand LPS on day 6, where indicated. Final read-out was performed after cell harvest on day 7. **(B)** Apoptosis rate of generated DCs was measured using a propidium iodide assay exploiting FACS. Nuclei fragmentation is indicated on the y-axis. **(C)** Surface expression of CD1a and CD14 was analyzed using FACS. **(D, E)** Modulation of co-stimulatory molecules was assessed. In **(D)** histograms for CD83 are depicted, with MFI and percentage of DCs in the top right corner for each condition. In **(E)** histograms are shown for CD40, CD54, CD80, CD83, CD86, DC sign. MFI of surface molecules from three to five representative experiments were pooled and are depicted. The significance was calculated according to the one-way ANOVA Dunnett or Tukey multiple comparison test and is related to the control group.

To elucidate potential immunomodulatory effects of ALKi on human DCs as most powerful antigen presenting cells (APC), we differentiated monocytes into moDCs in the presence of GM-CSF and IL-4. The ALKi crizotinib or alectinib were applied starting from day 0 on every other day, followed by final LPS maturation on day 6 **(** **Figure 1A** **)**. Chosen concentrations of ALKi correlated with patient´s serum concentrations.

We first excluded toxic effects of alectinib and crizotinib on moDCs. We found that neither crizotinib nor alectinib significantly increased nuclei fragmentation up to a concentration of 1 µM **(** **Figure 1B** **)**, indicating that the chosen ALKi concentration did not induce a significant amount of apoptosis.

We next aimed to analyze the effects of ALKi on DC phenotype. Usually, moDCs express low levels of the monocyte marker cluster of differentiation (CD)14 and upregulate CD1a, which is a typical DC marker and essential for the presentation of antigens. After stimulation, e.g. with a Toll like receptor (TLR)-ligand, costimulatory molecules will be uregulated. We here found that CD14 was not affected by neither alectinib nor crizotinib treatment, but CD1a was down-regulated by crizotinib treatment after lipopolysaccharide (LPS) maturation in a dose-dependent manner **(** **Figure 1C**, p=0,0156**)**. Moreover, upon (TLR)-ligation, crizotinib-treated moDCs showed a significantly reduced expression of the activation markers CD40, CD54, CD80, CD83, CD86 when compared to vehicle- or alectinib-exposed moDCs **(** **Figures 1D, E**, p-values are displayed in the figure**)**. No significant differences were detected for HLA-ABC and HLA-DR, indicating an unchanged antigen presentation, nor for CD155, CD47 and CD137L (not shown). Taken together, we were able to demonstrate that crizotinib profoundly suppresses the differentiation of monocytes to moDCs and reduces their capacity to upregulate DC activation and co-stimulatory markers. MoDCs with deficiencies in costimulation are known to induce inferior antigen-specific immune responses, thereby potentially reducing anti-tumor immunity.

### Crizotinib-treated moDCs show reduced migratory capacity

**Figure 2:**
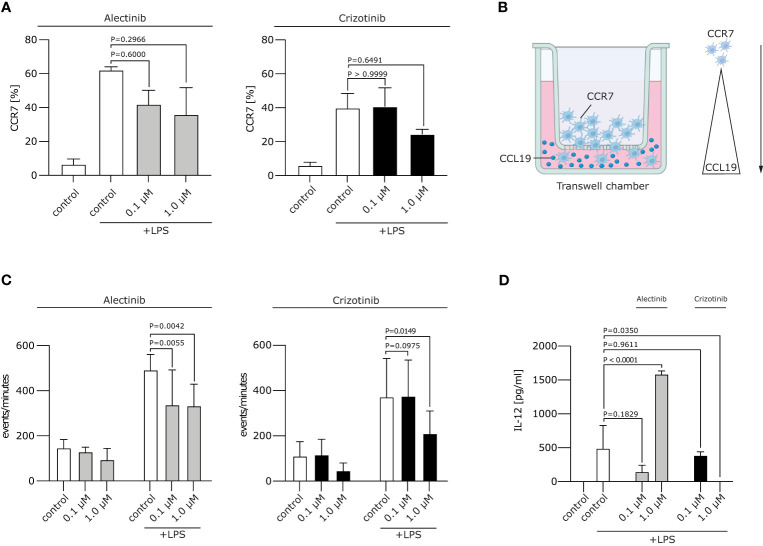
Crizotinib impairs the migratory behaviour and IL-12 production of moDCs. **(A)** Alectinib/Crizotinib-treated and LPS-stimulated human moDCs were assessed for their CCR7 expression using FACS. **(B)** Schematic experimental design for a migration assay along a CCL19 gradient. In brief, a transwell assay was performed. Generated DCs were put in the upper chamber, CCL19-containing medium in the lower chamber. **(C)** Alectinib/Crizotinib-treated and LPS-stimulated human moDCs were analyzed for their migratory behavior towards CCL19/MIP-3β in a transwell assays. Three to five representative experiments were pooled and significance was calculated according to 1-way ANOVA Dunnett's multiple comparison test and is related to the vehicle control. **(D)** MoDCs were generated as described in **(C)**. Il-12 was measured in supernatants exploiting ELISA. The significance was calculated according to the 1-way ANOVA Dunnett multiple comparison test and is related to the vehicle control.

We next wanted to analyze whether ALKi-treated moDCs display a stable migratory capacity. This is important in the context of DC migration to secondary lymphoid organs, where antigen presentation and T cell induction take place. Mature moDCs express chemokine CC motif receptor 7 (CCR7), the receptor for CCL19/MIP-3β, which guides DC transit from peripheral tissue to draining local lymph nodes following a CCL19/MIP-3β gradient. We found the typical upregulation of CCR7 on moDCs after LPS stimulation, but no significant changes in CCR7 expression after treatment with crizotinib or alectinib **(** **Figure 2A** **)**. Of note, migration of day 5 1 μM crizotinib-treated and LPS-matured human moDCs towards CCL19 was severely impaired **(** **Figures 2B, C**, p=0.0149**)** in comparison to alectinib-treated DCs. This indicates that 1 μM crizotinib treated DC migrate less efficiently. Given the unchanged CCR7 expression, CCR7 is most likely not explaining the observed reduction of DC migration. However, as described previously (22), a variety of other mechanisms in DCs can result in impaired DC migration.

### Crizotinib suppresses cytokine production by LPS-matured DCs

Upon activation, moDCs typically produce cytokines, especially Interleukin (IL-12), which are highly essential for the potent induction of anti-tumor immune responses. IL-12 is involved in the differentiation of naive T cells into Th1 cells, stimulates T cell growth, function and cytotoxic activity (23). We found that treatment of moDCs with crizotinib led to decrease of IL-12 production (p=0.035), while IL-12 was upregulated by alectinib (p<0.0001). This suggests that DC-driven T cell responses might even be improved by alectinib **(** **Figure 2D** **).**

## Discussion

The discovery of new genetic alterations and the approval of targeted therapies have impressively changed the therapeutic landscape of NSCLC and improved survival outcome (10). The clinical efficacy of cancer therapies relies on the induction and presence of anti-tumor immunity, which is predominantly characterized by tumor-specific T cells. For efficient CD8+ T cell induction, DCs have to take up tumor-derived antigens and present these on major histocompatibility complex (MHC) class I and II molecules in the presence of B7 molecules (20). Although a variety of approved ALKi exists, little is known on their potential immunomodulatory effects and anti-tumor immunity. ALKi have been shown to modulate the immunosuppressive microenvironment of ALK-mutated NSCLC by inducing upregulation of human leukocyte antigen (HLA), tumor-associated antigens and decreasing checkpoint molecule (16, 17, 24) expression, such as PD-L1 (16).

Due to the approval of ALKi as first line therapy^11^, many patients will receive immunotherapy upon progression on ALKi. Furthermore, new concepts are currently being tested which integrate targeted therapies into multimodal chemo- or immunotherapeutic treatment concepts (25–28). For both conditions, the immune constitution of a patient is of utmost importance for therapy responses and outcome.

Crizotinib has been impressively shown to induce immunogenic cell death. In combination with cisplatin, an increase in the expression of PD-1 and PD-L1 in tumors, coupled to a strong sensitization of NSCLC to checkpoint inhibitors (CPI) could be detected (14).

In our study, we elucidated whether ALKi exert immunosuppressive effects on human DCs as important players in anti-tumor immunity and questioned whether this immunosuppression differs among ALKi.

We found that DC differentiation and DC function is heavily impaired upon crizotinib exposure while alectinib displays no significant immunosuppressive effects. Further, differentiation of monocytes into moDCs was markedly impaired by crizotinib, which modulated proper DC differentiation and phenotype.

It has been shown previously that some TKIs, amongst crizotinib, can induce immunogenic cell death (14, 15) and activate the NLRP3 inflammasome in myeloid cells (19) which could partly contradict our data. Immunogenic cell death may unmask novel tumor antigens. However, for proper CD8+ T cell induction, these antigens must not only be available, but also be presented by DCs in the context of co-stimulatory molecules. In consequence, a reduced expression of these molecules due to crizotinib treatment might reduce the CD8+ T cell response. Regarding the inflammasome, it is not yet entirely clear whether inflammasome activation always promotes the upregulation of maturation signals in DCs (29). While some have reported that NOD-like receptors (NLRs) and TLR synergistically increase DC activation and functional properties of DCs upon reception of danger or infectious signals, in their absence a rather immunosuppressive DC state can be induced in DCs (30).

Moreover, we found that cells differentiated in the presence of crizotinib displayed reduced antigen uptake as well as co-stimulatory molecules such as CD83, CD80, CD86 upon TLR stimulation. The impaired DC function was further emphasized by a diminished migratory capacity of DCs and reduced secretion of IL-12 as important cytokine for T cell induction.

Taken together, we show that crizotinib, but not alectinib, modulates DC phenotype and profoundly suppresses DC function. The abovementioned immunostimulatory effects of ALKi in general, such as PD-L1 down-regulation, upregulation of HLA molecules, immunogenetic cell death and increased expression of tumor-associated antigens could further be improved by alcetinib which maintains DC functionality in contrast to crizotinib.

In consequence, using crizotinib could impair anti-tumor immunity, which is especially essential in the context of therapy combinations or follow-up treatments with immunotherapy targeting PD-1 or PD-L1.

Our data might also help to explain why more clinical infections occur under crizotinib treatment. Both aspects should be considered when ALKi are applied in patients as monotherapy or in multimodal treatment concepts.

### Limitations

We here provide evidence that crizotinib suppresses DCs, but not alectinib. DCs are important players in anti-tumor immunity, but represent only about 0.5% of all cells in the peripheral blood. This makes phenotypic and functional analyses of circulating DCs very difficult. We therefore decided to use human monocyte-derived DCs, which mirror ALKi-induced immunomodulation, but cannot replace *in vivo* analyses of TKI-treated patients. Moreover, other immune cell populations such as T cells, NK cells and myeloid cells should be investigated in detail.

### Future perspectives

In the era of personalized medicine, patients are treated according to the genetic fingerprint of their tumor. Nowadays, targeted and immunotherapeutic approaches are applied subsequently during the course of disease, but in future, a combination of both is very likely. If an ALKi such as crizotinib hampers immunity, the effect of combination treatments with e.g. an CPI might reduce efficacy. For this reason, the exact knowledge of ALKi-induced immunomodulation is important and should be taken into account.

## Data availability statement

The raw data supporting the conclusions of this article will be made available by the authors, without undue reservation.

## Ethics statement

The studies involving humans were approved by the institutional ethics committee of the University Hospital of Bonn (#173/09). The studies were conducted in accordance with the local legislation and institutional requirements. The participants provided their written informed consent to participate in this study.

## Author contributions

AH: Conceptualization, Data curation, Formal Analysis, Investigation, Methodology, Validation, Writing – original draft, Writing – review & editing. SH: Conceptualization, Formal Analysis, Methodology, Writing – review & editing. SD: Formal Analysis, Investigation, Methodology, Writing – review & editing. CF: Formal Analysis, Investigation, Methodology, Writing – review & editing. PB: Funding acquisition, Project administration, Resources, Supervision, Validation, Writing – review & editing.
